# EXPERIMENTAL ANALYSIS IN WISTAR RATS OF BONE GRAFTS PREPARED BY DIFFERENT STERILIZATION METHODS

**DOI:** 10.1590/1413-785220253303e289865

**Published:** 2025-08-18

**Authors:** Pedro Gabriel Minikowski Martins, Luan Praxedes de Oliveira, Thiago Mota dos Santos, André Vinícius Silva Müller, Erick Leonardo Martins Nascimento, Juliano Valerio Bortolletto

**Affiliations:** 1 Hospital e Maternidade Angelina Caron, Departamento de Ortopedia e Traumatologia Campina Grande do Sul, Parana, PR, Brazil.; 2 Clínica Bortolletto, Marechal Candido Rondon, Parana, PR, Brazil.

**Keywords:** Rats, Sterilization, Freezing, Irradiation, Ethylene Oxide, Proteoglycans, Ratos, Esterilização, Congelamento, Irradiação, Óxido de Etileno, Proteoglicanos

## Abstract

**Objectives:**

To evaluate *in vitro* the bone graft preparations sterilization (BG) by freezing, gamma irradiation and ethylene oxide and compare *in vivo* their osseointegration in Wistar rat femurs.

**Method:**

This basic controlled experimental study carried out in two phases, *in vitro*. First, *in vitro*, it was evaluated the sterilization of bone allografts exposed to *S. aureus*, *A. baumanii*, *M. tuberculosis* and *C. albicans*. In the second phase, *in vivo*, bone allografts osteointegration was compared in thirty-two Wistar rats, separated into four groups. In the control group (CG), fenestrations were carried out in the femur and not given a graft. Other groups received bone allografts prepared by freezing to –70ºC, ethylene oxide and 25KGy gamma irradiation. After thirty days, femurs were histologically evaluated by Hematoxylin-Eosin and Alcian Blue colorations and the Fisher's Exact test was used with p≤0.05.

**Results:**

In the *in vitro* study was observed lower level of decontamination by freezing method (26.6±21.09%) meanwhile and the other methods being 100% efficient. In the *in vivo* study, we observed no significant differences between the inflammatory reaction, mineralization and osteointegration. The percentage of proteoglycans was higher in the Gamma Irradiation Group and Ethylene Oxide Group. However, there were no significant differences. In the Frozen Graft Group, there was an absence in 37.5% of proteoglycans.

**Conclusion:**

The preparation of the bone allografts by freezing is less effective than the other methods and there were no differences in the osteointegration of the bone allografts in rat femurs.

## RESUMO

**Objetivos:**

Avaliar in vitro a esterilização das preparações de enxerto ósseo (EO) por congelamento, irradiação gama e óxido de etileno, e comparar in vivo sua osteointegração em fêmures de ratos Wistar.

**Método:**

Estudo experimental básico controlado realizado em duas fases. Primeiro, in vitro, foi avaliada a esterilização dos aloenxertos ósseos expostos a S. aureus, A. baumannii, M. tuberculosis e C. albicans. Na segunda fase, in vivo, comparou-se a osteointegração dos aloenxertos ósseos em trinta e dois ratos Wistar divididos em quatro grupos. No grupo controle (GC), foram realizadas fenestrações no fêmur sem enxerto. Os demais grupos receberam aloenxertos ósseos preparados por congelamento a –70ºC, óxido de etileno e irradiação gama de 25 kGy. Após trinta dias, os fêmures foram avaliados histologicamente com colorações Hematoxilina-Eosina e Azul de Alcian, e o teste exato de Fisher foi utilizado com p ≤ 0,05.

**Resultados:**

No estudo in vitro, observou-se menor nível de descontaminação pelo método de congelamento (26,6 ± 21,09%), enquanto os outros métodos apresentaram 100% de eficiência. No estudo in vivo, não foram observadas diferenças significativas entre os grupos quanto à reação inflamatória, mineralização e osteointegração. A porcentagem de proteoglicanos foi maior nos grupos Irradiação Gama e Óxido de Etileno; entretanto, as diferenças não foram estatisticamente significativas. No grupo de Enxerto Congelado, houve ausência de proteoglicanos em 37,5% dos casos.

**Conclusão:**

A preparação dos aloenxertos ósseos por congelamento é menos eficaz em termos de esterilização em comparação com os outros métodos. No entanto, não foram encontradas diferenças na osteointegração dos aloenxertos ósseos em fêmures de ratos.

## INTRODUCTION

The use of bone grafts from individuals of the same species (homologous) aims to repair skeletal defects while preserving their biological and mechanical characteristics.^1^ The use of homologous bone grafts has increased significantly in recent decades due to the development of new surgical techniques, the significant increase in high-energy trauma, an increase in the number of hip and knee arthroplasty revision surgeries, and, above all, the impossibility of obtaining large quantities of autologous bone grafts (from the individual themselves) have generated the need to improve bone reconstruction and replacement techniques.^2^ Dawson et al. in 1981 evaluated patients who underwent iliac bone graft removal and found that many reported pain or discomfort at the graft removal site years after surgery.^3^ The development of musculoskeletal tissue banks has broadened the prospects for implementing this therapeutic approach in orthopedics. Cited by Mozella et al.,^4^ the foundations of bone transplantation established by Ollier in 1867, through the recognition of the osteogenic properties of bone and periosteum, have had several contributions to date, including low-temperature storage methods proposed by Albee in 1912. Freeze-drying*,* recommended in 1951 by the United States Navy Tissue Bank*,* ^1^ the use of irradiation with Cobalt-60, and by Turner in 1956,^5^ and finally Friedlander in 1985,^6^ proved the beneficial influence of cold on the preservation of osteogenic characteristics. Currently, the influence that these bone graft processing methods have on bone tissue has been the subject of study. Autologous bone grafting is biologically the gold standard for bone transplants,^4^ and in some cases, when its use is limited, homologous bone grafting is the main alternative and can be used fresh, frozen at low temperatures, or freeze-dried. When used fresh, they induce an important immune response in the host, which can reduce their biological osteoinductive properties or even cause resorption of the transplanted bone tissue.^7^ When frozen at −70ºC, the most suitable preservation method for most orthopedic procedures, it reduces immunogenicity, inhibits bacterial growth, and keeps the graft in good condition for use, in addition to being technically accessible in several bone banks.^8^ Freeze-drying is also effective, but the mechanical strength of the graft is reduced, making its use as a structured bone segment contraindicated.^2^

The most important factor in musculoskeletal tissue transplantation is the safety of the recipient.^9^ Donor selection, proper collection techniques, and adequate processing and storage of grafts are mandatory and fundamental routines for their safety. However, some studies have shown contamination in bone graft samples, even with the application of these rigorous techniques. Due to this fact, many bone banks have used optional methods of sterilizing grafts as an additional measure to control the transmission of infectious diseases. This practice has been recognized as secondary sterilization, in which the most commonly used methods are irradiation with gamma rays obtained from Cobalt-60 and ethylene oxide, with no consensus yet on the nature and extent of its effects *in vivo*.^10^ This study aims to compare in vitro the effectiveness of sterilization by freezing, gamma irradiation, and exposure to ethylene oxide of experimentally contaminated homologous bone grafts and *in vivo* the osseointegration of these preparations in the femurs of Wistar rats.

## MATERIALS AND METHODS

### Experimental design

**Figure 1:**
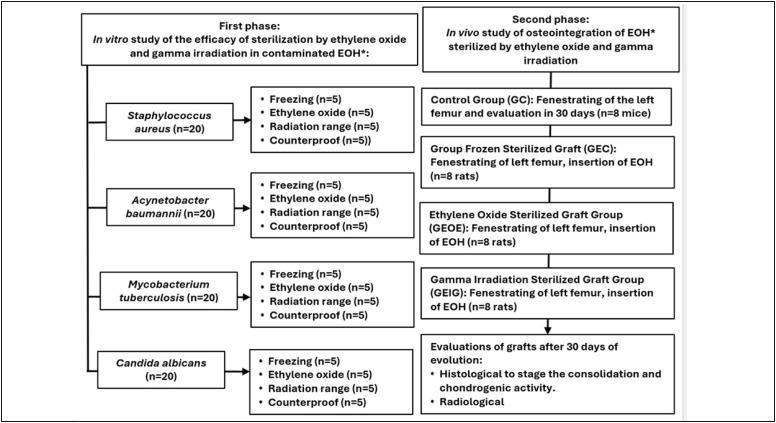
Diagram of the methodology adopted (*Homologous bone grafts).

This study was approved by the Animal Research Ethics Committee (CEPA) of the Angelina Caron Hospital in accordance with protocol no. 01/22 CEPA/HAC and carried out at the Animal Experimentation Center of the Angelina Caron Hospital and Maternity Ward in two phases. In the first phase, an *in vitro* study was conducted to compare bone graft sterilization techniques experimentally contaminated with different microorganisms. On the second day, an *in vivo* study was performed to assess whether different sterilization techniques could alter the osseointegration of bone grafts in Wistar rats. (Figure 1)

### Preparation of grafts

Grafts were prepared from the femurs of Wistar rats (*Rattus norvegicus albinus, Rodentia mammalia*), selected from different previous experiments in which there was no interference with bone tissue, at the HAC animal experimentation center. The grafts were prepared under strict aseptic conditions, under laminar flow, with all muscle tissue being resected and the femurs being fractionated into fragments of approximately 5 mm x 2 mm using a KG SORENSEN^®^ model 1022 diamond dental drill and stored in sterile jars. For the first phase of this study, 60 grafts were used, and in the second phase, 32 grafts were used. They were separated into groups of 15 grafts and sterilized as follows:

Ethylene Oxide Sterilization (GEOE): It was carried out in a chamber with a sterilizing mixture of 90% ethylene oxide (650 mg/liter) and 10%CO_2_, for three hours of exposure at a temperature of 50ºC, humidity of 40%, with mechanical aeration with compressed air pulses and nitrogen drag.

Gamma-irradiated allograft (GIA): Telecobaltotherapy (Cobalt-60) to receive 25KGy gamma irradiation, using Th780 (Theratronics^®^) equipment, as previously described by Herngou et al. in 1993^11^ and Nguyen et al. in 2007.^12^

Frozen bone graft (FBG): Freezing at –70º in a freezer model IULT 2005D (Indrel^®^). Subsequently, all grafts were kept at a temperature of –70ºC until ready for use.

### First Phase - In vitro study of the effectiveness of sterilization by freezing, ethylene oxide, and gamma irradiation

#### Microorganisms used

Strains of *Staphylococcus aureus*, *Acinetobacter baumanii*, and *Mycobacterium tuberculosis* bacteria *and* *Candida albicans* yeast *from the* HAC Microbiology Laboratory collection were used, isolated from clinical cases and identified by an automated method using BD Phoenix M50 equipment.

#### Contamination of grafts with bacteria and fungi

The bacterial inoculants to be used as contaminants were prepared by culturing the selected bacteria in brain and heart infusion broth (KASVI^®^ - K25-1400) for 24 hours at 37°C. The *Candida albicans* sample was cultured in tryptone soy broth (Himedia^®^ - AG6021). They were centrifuged for 10 minutes at 3,000 rpm and the supernatants were discarded. The microbial sediment obtained was resuspended in isotonic saline solution pH 7.4 and compared on the MacFarland scale in tube 6 corresponding to10^6^ CFU/ml. Next, batches of 15 bone grafts were submerged in each of the inoculants for 24 hours at room temperature, removed from the contaminating solutions, and placed in sterile containers. Ten grafts were selected from each group of contaminated grafts to undergo preparation by freezing at –70ºC, ethylene oxide, and gamma irradiation. Five remaining grafts were kept at 4 to 8°C and considered counterproof. After preparation, the grafts and counter-samples were submitted to culture and identification of contaminating bacteria and fungi by an automated method using BD Phoenix M50 equipment, and then the percentages of contaminated grafts were calculated.

### Second Phase – In vivo study of osteointegration of homologous bone grafts sterilized by ethylene oxide and gamma irradiation

#### Experimental environment

The rats were housed in groups of five in polypropylene boxes suitable for the species, in an environment with a controlled temperature between 19 and 22°C and automatically regulated light/dark cycles of 12 hours. They were fed species-specific feed (Nuvilab - Quimitia S.A.®) and water *ad libitum*. The waste was removed and the boxes were replaced every 24 hours, according to the standards of the animal facility where the study was conducted.

#### Animals used

Thirty-two male rats of the Wistar strain (*Rattus norvegicus albinus, Rodentia mammalia*) were used, obtained from the animal facility of the Universidade Federal do Paraná, weighing 213±9.2 g, separated into four groups of eight rats each.

### Surgical procedure

**Figure 2:**
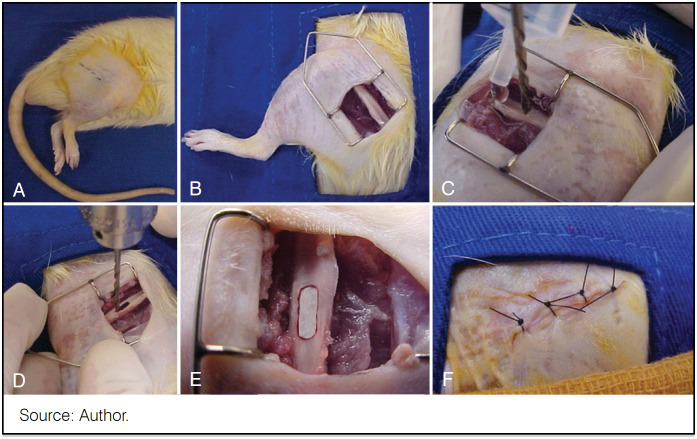
Illustrative diagram of the surgical procedure and bone graft implantation in Wistar rats. (A): trichotomy and degermation with iodized alcohol on the lateral side of the left hind limb thigh; (B): 2.0 cm incision in the skin, with subsequent incision of the fascia lata, the femur approached through the divulsion of the vastus lateralis and biceps femoris muscles; (C): with the aid of a sterile drill connected to a perforator; (D): three contiguous perforations of the cortical bone on the lateral aspect of the femoral diaphysis under irrigation with saline solution; (E): implantation of the bone graft; (F): approximation of the muscles and then suture of the skin with four separate simple stitches.

The surgical procedures were performed in an environment exclusively reserved for experimental surgeries, which was properly sanitized and air-conditioned. The animals were subjected to general anesthesia via intramuscular injection, with 75 mg/kg of Ketamine (Ketamin-Cristalia^®^) combined with 7.5 mg/kg of Xylazine (Calmiun-Agener União^®^), followed by trichotomy and degreasing with iodized alcohol on the lateral side of the left hind limb. With sterile instruments and surgical field, a 2.0 cm incision was made in the skin, followed by an incision of the *fascia lata*. The femur was approached by dissecting the vastus lateralis and biceps femoris muscles. With the aid of a sterile 2 mm diameter drill bit connected to a 1/2" GSB 16RE Bosch^®^ drill, three contiguous holes were drilled into the cortical bone on the lateral side of the femoral diaphysis, in its middle third, under irrigation with sterile saline solution, which together formed a bone fenestration measuring approximately 6×2 mm. Bone grafts were implanted in these bone fenestrations according to the groups planned in this study. After these procedures, the muscles were approximated and the skin was sutured with four simple separate stitches using 4.0 mononylon thread (Ethicon - Johnson-Johnson®), radiological evaluation, and then relocated to the animal facility where they received oral dipyrone diluted in water (90 mg/ml) for three days and maintained under the same preoperative conditions. (Figure 2)

### Radiological Assessment

All rats were X-rayed using Diagnost 15 MXG-Phillips^®^ Medical System equipment and Kodak^®^ 24×30 cm radiographic film to check for fractures and the location of fenestrations.

### Histological Evaluation

On the 30th day, the animals received a lethal dose of 300 mg/kg of sodium thiopental (Cristália^®^) intraperitoneally, thereby inducing death by cardiorespiratory arrest. The left hind limbs were then disarticulated and the femurs were dissected, preserving the soft tissue surrounding the bone callus of the graft. Next, the pieces were subjected to histological evaluation using 10% formaldehyde fixation, decalcification in 5% nitric acid, dehydration in alcohol, and inclusion in paraffin. Sequential 5µm transverse histological sections of trabecular bone tissue were prepared using Harris Hematoxylin and Eosin (HE) and Alcian Blue (AB) stains. The analysis was performed under optical microscopy (Zeiss^®^ microscope, Axiostar Plus), and the stages of bone graft consolidation were determined by two pathologists in random sequence of the slides, without knowledge of the group to which the animals belonged. The methodology proposed by Mainardes and colleagues in 2007^13^ was used, with sections stained with HE and the adoption of histological criteria that organize the bone consolidation process into four phases. 1) fibroblastic, 2) cartilaginous or collagenous, 3) osteogenic phase, 4) ossification with immature bone or remodeling. These criteria were associated with the proposal by Allen et al.,^14^ according to a five-point scale (Table 1) as parameters for classifying the graft consolidation phase. Alcian Blue (AB) staining was used to evaluate proteoglycans, which reflect chondrogenic activity, classified as absent, mild, moderate, or abundant. The absence of proteoglycans reflects low chondrogenic activity in mature bone callus, and abundant chondrogenic activity, indicated by high amounts of proteoglycans, reflects tissue repair in the inflammatory phase.^14^

**Table 1:** Description of parameters for classifying the fracture consolidation phase according to Allen et al. 1980.^14^

Bone repair stage	Histological findings
Grade 4	Complete bone union
Grade 3	Incomplete bone union with presence of cartilage remnants in the callus
Grade 2	Complete cartilaginous union with a cartilaginous bridge connecting the bone ends
Grade 1	Incomplete cartilaginous union with presence of fibrous tissue remnants and cartilaginous plate
Grade 0	Pseudarthrosis

### Statistical analysis

For statistical comparisons, contingency tables were used with Fisher's exact test, with a significance level of 0.05 for rejection of the null hypothesis.

## RESULTS

### Results of an in vitro study of the percentages of decontamination of bone grafts by freezing, ethylene oxide, and gamma irradiation

**Figure 3:**
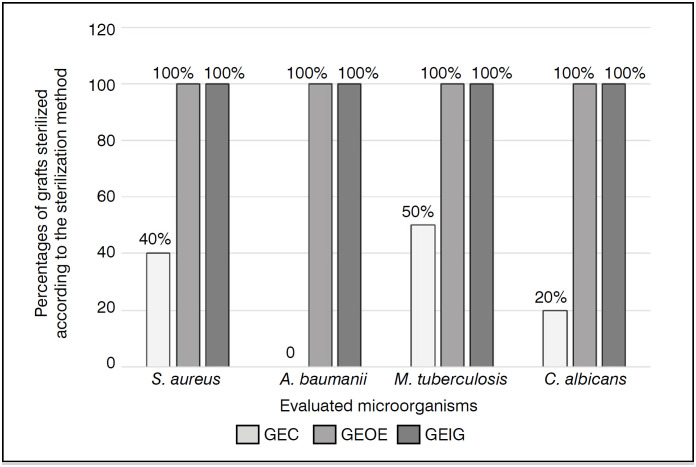
Statement of bone graft decontamination percentages according to the microorganism tested.

All counter-evidence from experimentally contaminated grafts demonstrated growth of the respective contaminating agents. As shown in Figure 3, there was a significant difference (p<0.001) between the percentages of decontamination of grafts after contamination with 10^6^ CFU/ml of *Staphylococcus aureus*, *Acynetobacter baumanii*, *Mycobacterium tuberculosis* and *Candida albicans*. It is observed that the lowest level of decontamination was obtained by the freezing method (26.6±21.09%). There was no difference between the use of ethylene oxide or gamma irradiation, both being 100% effective. As for the effectiveness of decontamination in relation to the microorganisms evaluated, Figure 3 shows that ethylene oxide and gamma irradiation sterilization methods were 100% effective against all bacteria and yeast evaluated. However, freezing had no bactericidal effect on *Acinetobacter baumanii*, and in 20% of grafts on *Candida albicans*, 40% on *Staphylococcus aureus*, and 46.7% on *Mycobacterium tuberculosis*.

### Results of an in vivo study of the osseointegration of bone grafts prepared by freezing, ethylene oxide, and gamma irradiation

There were no deaths in rats subjected to homologous bone grafts during the thirty days of evolution. The rats in the control group, which underwent bone fenestration without receiving a graft, showed an area of abrupt discontinuity of the cortical layer in the histopathological evaluation by HE staining and abundant amounts of proteoglycans.

### Evaluation of the osseointegration process of homologous grafts

**Figure 4:**
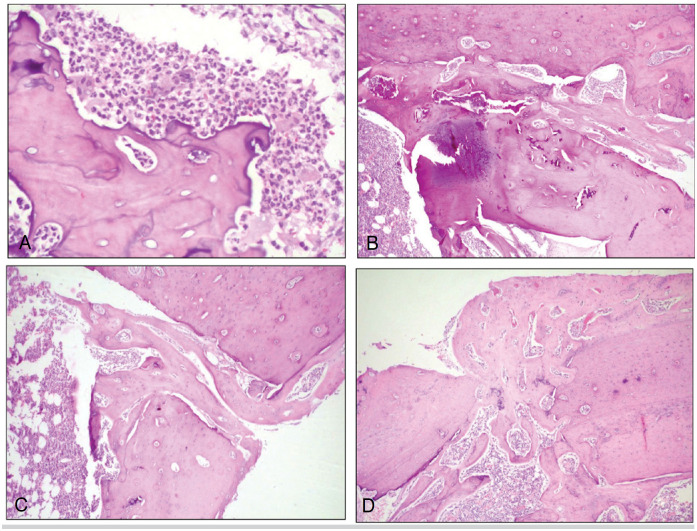
Photomicrographs of histological sections of homologous bone grafts from rat femurs. (A): pyogranulomatous inflammatory reaction around bone graft fragment (H.E. 200x). (B): areas of mineralization in the bone graft (H.E. 100x). (C): formation of bone "bridges" by bone callus between the graft and the original bone tissue (H.E. 100x). (D): bone callus in the ossification phase (H.E. 100x).

The classification of the consolidation process of homologous grafts performed on histological sections of the grafted area by HE staining (Figure 4) is shown in Table 2, the classification of the graft consolidation phase in Figure 7, and the amount of proteoglycans in Figure 4.

**Table 2:** Percentage classification of histopathological findings by HE staining of the grafted area in rat femurs regarding inflammatory reaction, mineralization, and osseointegration.

Histopathological Evaluations FBG		Percentages of histological findings according to groups		
		GIA	GEOE	
Inflammatory Response	Absent	28.58%	12.50%	0
	Present	71.42%	87.50%	100%
Mineralization	Absent	71.42%	62.25%	83.34%
	Present	28.58%	37.75%	16.66%
Osseointegration	Absent	28.52%	37.50%	0
	Present	71.42%	62.50%	100%

Table 2 shows that the group of rats that received ethylene oxide-sterilized homologous grafts (GEOE) presented a higher level of inflammatory reaction (100%), predominantly mild (50%), and also presented a lower percentage of mineralization (16.66%) and osseointegration in 100%. The group that received gamma-irradiated allograft (GIA) presented an inflammatory reaction in 82.50% of cases, with the intense type prevailing (37.5%). Bone mineralization was absent in 62.25% and osseointegration occurred in half of the rats (37.5%). The group of rats that received frozen bone graft (FBG) showed a 71.42% inflammatory reaction, 57.14% of which was mild, bone mineralization in 28.58%, and osseointegration in 71.42%. There were no significant differences between these findings.

**Figure 5:**
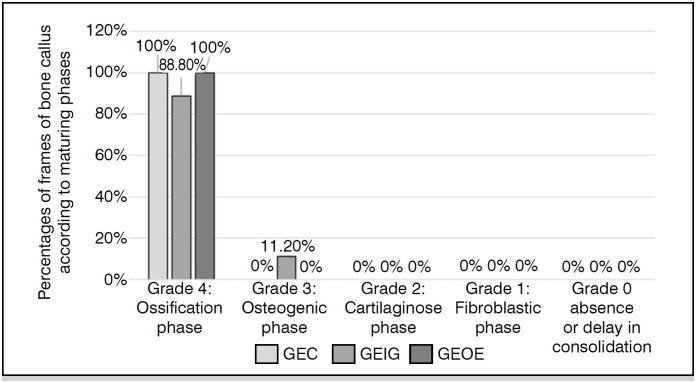
Statement of the graft consolidation phase classification.

Figure 5 shows that the FBG and GEOE groups demonstrated the same behavior in relation to graft consolidation, as both presented 100% in the ossification phase corresponding to grade 4 (complete bone union). The GIA group presented 88.8% in phase 4 and 11.2% in osteogenic phase – grade 3 (incomplete bone union). There were no significant differences between these findings.

**Figure 6:**
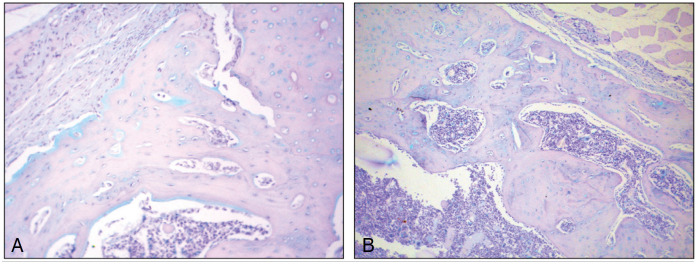
Photomicrographs showing bone callus in the ossification phase with a slight amount of proteoglycans (Alcian Blue). 100x).

**Figure 7:**
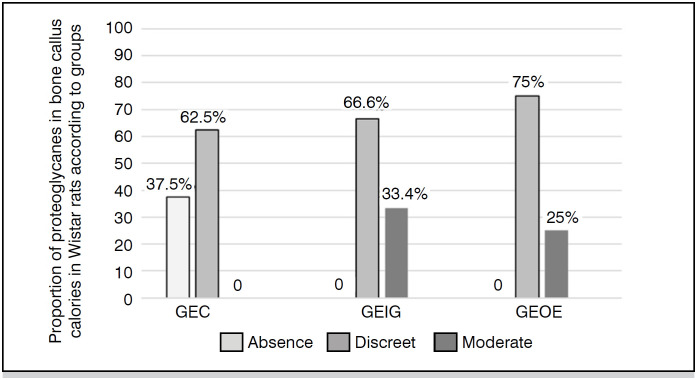
Statement of the percentage quantification of proteoglycans.

In the percentage quantification of proteoglycans, whose high quantity is related to abundant chondrogenic activity and reflects tissue repair still in the inflammatory phase (Figure 6), the FBG group stood out with 37.5% absence of proteoglycans, thus the highest percentage of low chondrogenic activity in mature bone calluses. The GIA and GEOE groups showed 33.4% and 25% moderate amounts of proteoglycans and 66.6% and 75% mild amounts, respectively (Figure 7 and Table 3), thus revealing greater amounts of cartilage tissue than bone tissue in the graft regions, but there were no significant differences between these groups.

**Table 3:** Statistical evaluation by Fisher's exact test of the presence of proteoglycans.

Comparative aspects	Groups compared	p-value
Presence of Proteoglycans	FBG x GIA	0.0824
	FBG x GEOE	0.0824
	GIA x GEOE	0.2

**Figure 8:**
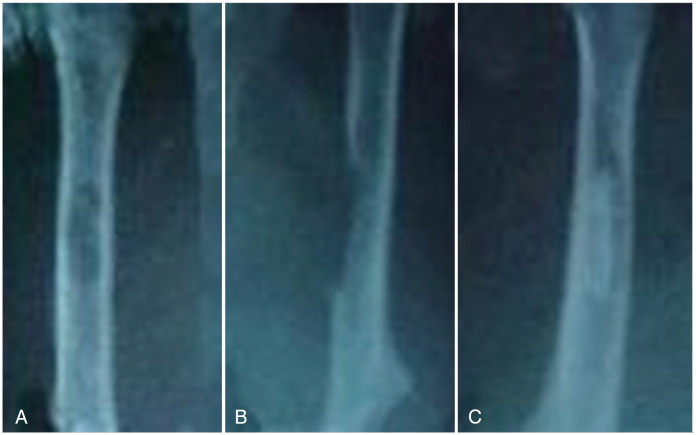
Radiographic images of rat femurs with 30 days of evolution. (A and B): Anterior-posterior and profile views of the fenestration in the left femur of the control group. (C): anteroposterior view showing homologous grafts.

Figure 8 shows radiographic images of rat femurs, where the absence of graft can be observed in the control group and the presence of graft in another group, as models.

## DISCUSSION

Based on the possibility that homologous grafts present a risk of transmitting infectious diseases,^15^ the present study proceeded in the first phase with an *in vitro* evaluation of the microbicidal capacity of freezing procedures at –70ºC, gamma irradiation at 25kGy, and exposure to ethylene oxide. For this purpose, groups of homologous grafts were separately contaminated with solutions containing 10^6^ CFU/ml of *Staphylococcus aureus*, *Acinetobacter baumannii*, *Mycobacterium tuberculosis*, and *Candida albicans* yeast, microorganisms commonly involved in severe infections. It was observed that the best results were obtained by gamma irradiation and ethylene oxide exposure procedures, achieving 100% sterilization of the grafts (Figure 3). Freezing at –70ºC, although presenting the lowest costs in the present study, contrary to the results obtained by Friedlander et al. in 1998,^16^ which demonstrate bactericidal action with the use of freezing at –70ºC, had no effect on *Acinetobacter baumanii*, and in 20% of grafts on *Candida albicans*, in 40% of *Staphylococcus aureus* and in 46.7% of *Mycobacterium tuberculosis* (Figure 3). This highlights the need to implement studies on current methods of managing homologous bone grafts, especially regarding freezing at –70ºC.

The second phase of this study aimed to verify in vivo the effects of freezing at −70ºC, gamma irradiation, and exposure to ethylene oxide on the osseointegration of homologous grafts. Rats (Rattus norvegicus) were chosen because they are widely used in scientific research due to their small size, which allows them to be available in statistically adequate numbers. They also present the ease of obtaining and housing, as well as their suitability for specific laboratory environments and affordable costs. The experimental model used in this study was based on the methodology proposed by Galia et al. in 2005,^1^ who compared the osseointegration of freeze-dried or frozen homologous and heterologous grafts in fenestrations produced in the femurs of Wistar rats. They used inflammatory cell count and osseointegration through bone neoformation, occurrence of fibrosis, and necrosis in bone grafts as indicators. Ideally, methods for preserving homologous grafts should reduce or eliminate the immunogenicity of homologous transplants, maintain osteoinductive factors, and preserve conditions for revascularization stimulation. Freezing between −70º and −80ºC is a method that offers these conditions, as well as practicality and maintenance of mechanical characteristics. In the present study, as shown in Table 2, freezing at –70º resulted in a lower percentage of inflammatory reaction in rats receiving these grafts compared to preparations by gamma irradiation and ethylene oxide, although not statistically significant (p=1), 71.42%, 88.5%, and 100%, respectively. As for the organization of the bone consolidation process, 100% of the rats that received frozen grafts presented grade 4, referring to complete bone union. In this regard, the preparation of grafts by freezing did not differ from other preparations, as ethylene oxide showed the same results and gamma irradiation showed 88.8% in grade 4 and 11.2% in grade 3 (Figure 5). As for the quantification of proteoglycans, in the control group where no bone grafts were inserted, it can be observed that all had abundant amounts of proteoglycans, indicating that the rats had bone regeneration capacity. Proteoglycans reflect chondrogenic activity, and freezing demonstrated their absence in 37.5% of rats and their presence in small quantities in 62.5%. The other preparations presented higher amounts, revealing less ossification in the grafts.

Aghaloo et al. in 2006^17^ showed decreased expression of osteoinductive factors PDGF, bFGF, and TGF-beta in irradiated rabbit tibiae compared to non-irradiated tibiae. These effects are proportionally dose-dependent. In the present study, a dose of 25 KGy was used, which is currently used by most bone banks.^11,12^

Munting et al. in1988^18^ showed that irradiation of bone grafts at a dose of 25KGy results in a 50% reduction in their osteoinductive capacity. In the present study, rats subjected to grafting prepared by gamma irradiation showed lower percentages of osseointegration (62.5%) (Table 2), confirmed by the presence of osteogenic phase (incomplete bone union) in 11.2% of cases (Figure 7) and discrete and moderate amounts of proteoglycans (Figure 7). These results suggest that these rats had greater amounts of cartilage tissue than bone tissue in the graft region. Regarding the use of gamma irradiation, studies by Lietman et al. in 2000,^19^ are noteworthy^,^ as they observed lower incidence of infection in irradiated grafts compared to non-irradiated grafts, but greater susceptibility to fractures.

Ethylene oxide is an effective sterilizing agent. This gas has the disadvantage of leaving residues in the graft due to chemical reactions that occur in the presence of water, which is characteristic of frozen grafts. It has high toxicity potential, as it is carcinogenic, mutagenic, and teratogenic, requiring adequate washing of the graft and a well-ventilated environment to dissipate residues after the application cycle. Its high residual concentration leads to an exuberant inflammatory reaction, which may impair the osseointegration of the homologous graft.^2,10^ This fact was confirmed in the present study, as grafts prepared with ethylene oxide showed higher percentages of inflammatory reaction (Table 2), but without alteration of osseointegration (Figure 7). The biological changes in bone grafts caused by sterilization with ethylene oxide are controversial. In this context, Arizono et al. in 1994^20^ demonstrated adverse effects of ethylene oxide on the osteoinduction process, the main deleterious factors being: the temperature at which the bones are sterilized, the time they are exposed to aeration, and the presence of ethylene oxide residues after sterilization.

## CONCLUSIONS

The *in vitro* evaluation of sterilization by freezing EOSA preparations showed lower efficacy than sterilization methods using gamma irradiation or exposure to ethylene oxide. There were no differences in the osseointegration of bone grafts sterilized by freezing, gamma irradiation, or exposure to ethylene oxide in the femurs of Wistar rats thirty days after bone grafting.
